# Method-Dependent Bond Strength of Thin-Bed Mortars with Coded Modifiers on Autoclaved Aerated Concrete

**DOI:** 10.3390/ma19214629

**Published:** 2026-10-31

**Authors:** Beata Łaźniewska-Piekarczyk, Adam Piekarczyk

**Affiliations:** 1 Department of Building Processes and Building Physics, Faculty of Civil Engineering, Silesian University of Technology, Akademicka 5, 44-100 Gliwice, Poland; 2 Department of Building Structures, Faculty of Civil Engineering, Silesian University of Technology, Akademicka 5, 44-100 Gliwice, Poland; adam.piekarczyk@polsl.pl

**Keywords:** autoclaved aerated concrete, masonry bond, thin-bed mortar, 1 mm bed joint, direct axial tension, bond-wrench test, admixture dosage, stress distribution

## Abstract

**Highlights:**

- The AAC bed-joint thickness was measured as 1 mm throughout, below the matching product profile’s 3 mm maximum;
- Public product documentation specifies the mortar type and use limits but does not verify the tested production batch;
- The 94 mm core sampled 2.92 times the bonded area of the preliminary 55 mm cores;
- Axial and bond-wrench tests produced different modifier rankings; individual data and exploratory models are reported.

**Abstract:**

This study reanalyzes the tensile bond of white-cement thin-bed mortar joints with a measured thickness of 1 mm in autoclaved aerated concrete (AAC), using axial core tension, the EN 1052-5 bond-wrench method, and companion hardened-mortar tests. Individual observations are summarized descriptively alongside exploratory formulation-mean models. For 94 mm cores, mixture C (0.229 wt.%) had the highest axial mean (0.528 ± 0.081 MPa; n = 4), versus 0.378 ± 0.065 MPa for the reference (n = 5). Mixture A had a low axial mean (0.139 ± 0.123 MPa) but the largest bond-wrench mean (0.351 MPa), demonstrating method-dependent rankings. The available product card for the C-coded powder describes it only as a re-dispersible synthetic-resin powder; the remaining modifiers are known only from broad recorded labels. Water carried by liquid O was included in the total mixing-water dose of 5.75 L per 25 kg of dry base mortar; however, its separate amount was not recorded. The 94 mm bonded area was 2.92 times that of the preliminary 55 mm cores, whose high scatter and unresolved data inconsistency preclude comparison. One wrench assembly was tested per mixture; incomplete fresh-state and conditioning records constrain inference. The study provides a method-aware comparison for this specific thin AAC joint without claiming novelty with respect to AAC bond research.

## 1. Introduction

Thin-bed mortar forms the load-transfer layer between autoclaved aerated concrete (AAC) units. Its small thickness does not make its mechanical role negligible: contact continuity, surface texture, water exchange at the interface, and the first minutes of mortar placement can determine whether the joint transfers tension and shear uniformly or through a limited set of contact patches. The measured bond, therefore, belongs to the mortar–unit system, not to the mortar alone. AAC bond studies have compared joint materials and mortar thicknesses and have shown that measured strength varies with test configuration and loading conditions [1,2,3]. A result from one geometry should, consequently, be interpreted as a response under that geometry rather than as a universal interface constant.

Research on thin-layer polymer–cement masonry is also relevant. Thamboo, Dhanasekar, and Yan assessed flexural and shear bond in 1–4 mm polymer–cement joints, while subsequent work examined curing and age [4,5]. Related mortar studies show that cellulose ethers and other polymers can alter water retention, consistency, pore structure, and hardened strength [6,7,8,9,10]. Ramdani et al. tested AAC masonry with a poly(vinyl acetate) (PVAc)-modified thin-bed mortar. At 28 days, they reported mean splitting-tensile strengths of 0.265 MPa for commercial mortar and 0.326 MPa for the mixture containing 15% PVAc relative to cement mass, an increase of about 23% [11]. This is an assembly splitting result, not a direct isolated measurement of tensile adhesion across the AAC–mortar interface; its dosage and test configuration also differ from the present low-dose, dual-method study. Their 2024 study reported improved workability and slower hardening at a 15% PVAc addition but did not report direct tensile adhesion across the AAC–mortar interface [12]. These studies show that modifier formulation can affect masonry response and fresh properties; however, they do not identify the coded additions in the present study.

The present study examines a white-cement AAC bed joint measured at 1 mm throughout, using two distinct loading approaches: direct axial tension on bonded cores and the EN 1052-5 [13] bond-wrench method on small masonry assemblies. The study also considers reported density and strength of companion mortar specimens and fits descriptive models to formulation means. Its contribution is deliberately narrow: it brings together a low-dose modifier screen, two unlike stress fields, and transparent data-level analysis for this particular material configuration. As far as the literature reviewed for this manuscript indicates, the exact combination is not represented in the cited AAC bond or thin-layer masonry papers. This contextual claim is not a claim of first-ever research on AAC bond or thin-layer mortars.

The 1 mm joint is a particularly sensitive scale for fresh-state variation. A small change in water demand, spread, or open time can determine whether the trowelled mortar wets the AAC surface and maintains contact across the bed. AAC also has a porous, suction-active surface; thus, water removal from fresh mortar can occur rapidly and may alter local hydration and interface formation. Since no flow value, open time, block moisture content, or surface absorption was recorded, these mechanisms provide a rationale for careful interpretation rather than a retrospective explanation of any individual result.

Therefore, the research questions were: (i) how the coded modifiers compare with the reference under each loading method; (ii) whether axial and wrench rankings agree when their different stress distributions are respected; and (iii) whether the mortar density and strength means explain the bond pattern at the formulation level. These questions are answered descriptively. The manuscript reports the experimental measurements directly and separates them from model sensitivities calculated during reanalysis.

## 2. Materials and Methods

### 2.1. Materials and Mixture Design

The masonry units were AAC blocks in density class 600, with a declared dry density of 575 ± 25 kg/m^3^, declared mean compressive strength of 3.0 MPa, and nominal dimensions of 180 mm × 240 mm × 590 mm. The mortar was described as a white-cement, thin-bed masonry mortar with a strength class of M5. M5 denotes the mortar’s declared compressive-strength class. A public profile for a matching Type T product identifies a dry mix of hydraulic binders, selected mineral aggregates, and admixtures; it specifies 5.75 L of water per 25 kg, a maximum bed-joint thickness of 3 mm, and an aggregate size below 0.8 mm [14]. This profile is a product context; the tested product/lot identifier was not retained. All reference and modified formulations used 5.75 L of total mixing water per 25 kg of dry base mortar. No extra water was added to compensate for reduced workability or to match fresh consistency. For O, water carried by the liquid addition was included in this 5.75 L total and was not added on top. The amount contributed by O was not recorded separately; thus, separately added water cannot be reconstructed. A public-safety datasheet listing for product type 0.1 is available [15]; however, neither public record identifies the tested batch or verifies its exact composition. EN 998-2:2016 covers factory-made masonry mortars, including thin-layer mortars [16]; no declaration of performance for the tested batch was available.

The modifiers were identified by short codes and broad chemical descriptions. Table 1 provides the documented descriptions without manufacturer or trade names. The product card associated with the C-coded powder describes it as a re-dispersible powder based on synthetic resin; it does not disclose the precise polymer family, monomers, auxiliary ingredients, or active-solids content [17]. C.0 and C are two dosage levels of this powder. M is identified as methylcellulose, a cellulose ether; however, its grade, molecular mass, and substitution characteristics are unknown. P is labelled “M-polymer acrylic,” supporting only an acrylic-based family; its exact polymer type and active-solids content are unknown. A is labelled “vinyl alcohol”, which does not by itself verify polyvinyl alcohol (PVA) identity or grade. O is labelled “vinyl acetate” and was used as a liquid; the description does not distinguish a monomer from a polymer dispersion or disclose its solids and carrier composition. Product/lot identifiers and complete additive formulations were not retained. Experimental notes state that C.0 and M.0 were reduced from 1.000 wt.% after poor workability was observed.

The manufacturer’s public archive lists a safety datasheet for product type 0.1 with a file date of 28 May 2026; the product profile and archive listing were consulted on 22 October 2026 [14,15]. These documents are retained as a public product context only. Because the product/lot identifier for the tested mortar was not retained, the records do not establish the composition of the batch used in the experiments.

Dosage is relative to dry mortar mass. Equivalents are mass conversions only; volume fractions cannot be calculated because component densities and active-solids contents are unknown. Available test notes indicate that C.0 and M.0 at 1.000 wt.% had poor workability.

The modifier quantities were expressed as a percentage of dry mortar mass. A dose of 0.229 wt.% corresponds to 2.29 g/kg or 57.25 g per 25 kg batch; 0.687 wt.% corresponds to 6.87 g/kg or 171.75 g per 25 kg batch; and 1.000 wt.% corresponds to 10.0 g/kg or 250 g per 25 kg batch. These are dry-mass conversions only. The cement fraction of the tested batch, additive density, active-solids content, volumetric dose, and separate amount of water carried by O were not retained; therefore, conversion to cement-mass percentage or active-polymer dose is not possible.

### 2.2. Specimen Preparation and Conditioning

Three specimen configurations were used. First, 55 mm-diameter AAC cores joined with mortar were prepared as a preliminary screen, with five specimens planned for R, C.0, M.0, P, and A. Second, 94 mm cores were prepared for axial tension, with six planned per mixture for R, C.0, C, M, P, and A. Mortar was applied to the AAC surface with a toothed trowel; the tooth dimensions and application path were not recorded. The bed-joint thickness was measured as 1 mm throughout. The measurement instrument and exact locations were not recorded. The matching product profile permits a maximum bed-joint thickness of 3 mm and specifies an aggregate size below 0.8 mm; this product limit is distinct from the measured 1 mm joint [14]. The 55 mm and 94 mm bonded areas were 2376 and 6940 mm^2^, respectively. After bonding, core assemblies were held under approximately 5 kPa compressive stress normal to the joint.

For the 94 mm core series, the bonded area of 6940 mm^2^ is consistent with the circular area calculated from the specified nominal diameter (πd^2^/4 ≈ 6939.8 mm^2^); the 55 mm area is approximately 2375.8 mm^2^. These values are nominal geometric areas and do not represent a measurement of the fraction in full contact. The joint thickness was measured as 1 mm throughout; however, individual location-by-location readings and measurement details were not recorded. No spatial thickness distribution or estimate of within-joint variation could, therefore, be calculated.

The third configuration was a masonry assembly made from six AAC half-blocks for the bond-wrench test. For each mixture, five successive bed joints were measured sequentially in one masonry assembly (one wall) as the blocks were detached. These are repeated joint-level readings within one assembly, not independent replicates; one assembly was tested per mixture. Approximately 5 kPa normal compression was applied to the joints after construction. Wrench results are, therefore, reported descriptively, with no between-assembly variability estimable from this design.

Core specimens damaged before testing or failing away from the mortar–AAC interface were excluded from the reported valid bond-strength series. The retained 94 mm counts are consequently below the planned six for R, C, and P, and for A; C.0 and M retained six. The exact failure classification for each excluded specimen was not recorded; thus, the exclusions are presented without reconstructing unrecorded failure causes.

The masonry assemblies and modifier-containing mortar specimens were kept in the same curing environment, with the same temperature and relative humidity. Numerical temperature and humidity values, and the exact elapsed time from mixing to assembly and testing, were not recorded. The masonry assemblies were tested after approximately 28 days; exact testing ages and the ages of companion mortar prisms were not stated. No water-absorption conditioning or moisture-equilibration step for the AAC surface was documented. These gaps matter because a small bed joint couples mortar hydration to the moisture state of the adjacent AAC.

### 2.3. Axial Tensile Bond Test

The core procedure was a project-specific direct axial tensile test, not a conventional small-dolly pull-off test. No standardized AAC bond method was identified in the available procedure description. Steel loading plates were bonded axially to the AAC core assembly using an epoxy-based adhesive. Tensile force was applied manually by a screw mechanism and measured with a strain-gauge load cell. Nominal axial bond strength was calculated as the maximum tensile force divided by the bonded area:

This test route was selected to load a larger portion of the AAC–mortar interface than a small-dolly pull-off measurement. Local pull-off tests on heterogeneous porous substrates can be strongly affected by the small sampled area, local pores or defects, core-edge damage, and departures of the crack path from the intended interface [18,19,20]. In this study, such a localized pull-off configuration was not used because the small detachment area was considered liable to distort representation of the bond across the thin joint. The 94 mm core increases the sampled area but does not remove the need to control eccentricity, preparation damage, or failure location.

f_t_ = F_max_/A_β_

Here, f_t_ is the nominal axial bond strength, F_max_ is the maximum recorded tensile force, and A_β_ is the bonded area. The adopted 94 mm cores provided A_β_ = 6940 mm^2^; the preliminary 55 mm cores provided 2376 mm^2^. A numerical loading rate, alignment tolerance, and measured eccentricity are not reported. EN 1542 is a pull-off test standard for products and systems on concrete and was not applied to these AAC core tests [18]. Methods in the literature note that localized pull-off measurements can be sensitive to substrate heterogeneity, eccentricity, coring damage, and failure outside the target interface [19,20].

### 2.4. Bond-Wrench Test

The masonry bond-wrench test followed EN 1052-5:2005 [13]. In the reported setup, load F_1_ acted through a lever arm e_1_ = 1020 mm; lever self-weight was represented by F_2_ = 450 N at e_2_ = 260 mm. The calculation used b = 295 mm for the tested section width and t = 180 mm for the block depth in the section. This t is a block dimension in the bending calculation, not the 1 mm bed-joint thickness. The symbol g denotes the weight of the portion detached above the tested joint. The corresponding edge-stress expression was:

f_w = [6(F_1_e_1_ + F_2_e_2_) − 4t(F_1_ + F_2_ + 0.25 g)]/(bt^2^).

The wrench produces a bending moment at the tested joint. Under an ideal elastic-section model, normal stress varies approximately linearly across the section, with tension at one edge and compression at the opposite edge. The calculated f_w is, therefore, an edge tensile stress derived from the lever geometry; it is not an area-average axial stress. All wrench-test failures are reported at the mortar–AAC interface. The loading rate was not reported.

The wrench configuration has different mechanics. The lever converts the applied force into a bending moment at the tested bed joint. Under the elastic rectangular-section idealization, the stress varies linearly across the section: tensile stress reaches its maximum at one edge, falls to zero at the neutral axis, and becomes compressive toward the opposite edge. The calculated value is, therefore, an edge stress under the method’s bending model. The axial test instead divides force by the full circular bonded area and assumes a nominally uniform mean stress. As a result, mixture A can rank low in direct tension yet high in wrench loading without an arithmetic contradiction: the two tests mobilize different portions of the interface and impose different stress gradients. The finding argues for reporting the method alongside any quoted bond strength.

The lever force and its self-weight act through different moment arms, while the detached upper portion contributes a gravity correction. In the nominal section model, the applied moment is balanced by a linear tensile-to-compressive stress distribution across the block depth. The bond plane is only 1 mm thick, but it is not the dimension used to calculate the section modulus: the parameter t = 180 mm is the block depth. Using the joint thickness in place of t would produce a physically incorrect stress calculation. Conversely, the calculated edge stress is not the mean stress across the bond area, because one edge is in tension and the opposite edge is in compression. It should not be compared numerically with the axial F/A value as if both measured the same material parameter.

### 2.5. Hardened Mortar Properties

Density and flexural and compressive strengths of hardened mortar were determined using the procedure reported as PN-EN 1015-11:2020 [21], the Polish adoption corresponding to EN 1015-11:2019 [21]. Mortar specimens containing modifiers were kept in the same curing environment as the masonry assemblies. The numerical temperature and relative humidity, test age, replicate counts for each property, and whether density refers to dry or conditioned mass are not reported. Section 3.4 presents the recorded tabulated means and standard deviations. A separate narrative summary provides reference flexural and compressive strengths of 2.22 and 6.50 MPa, whereas the table provides 1.77 and 7.05 MPa; the tabulated values are used consistently here and the discrepancy remains unresolved.

The experimental notes state that mixture preparation followed EN 196-1:2016 [22]. This standard is a cement strength-testing method; it does not, by itself, specify the complete batching protocol for a modified factory-made thin-bed mortar. The mixing procedure is, therefore, described only to the extent documented, without claiming a verified standardized protocol for the modifier additions. The same notes identify PN-EN 1015-11:2020 for hardened-mortar flexural and compressive strength. These recorded hardened properties are used as companion responses, not as direct measurements of bond.

### 2.6. Data Handling and Statistical Scope

**Figure 1:**
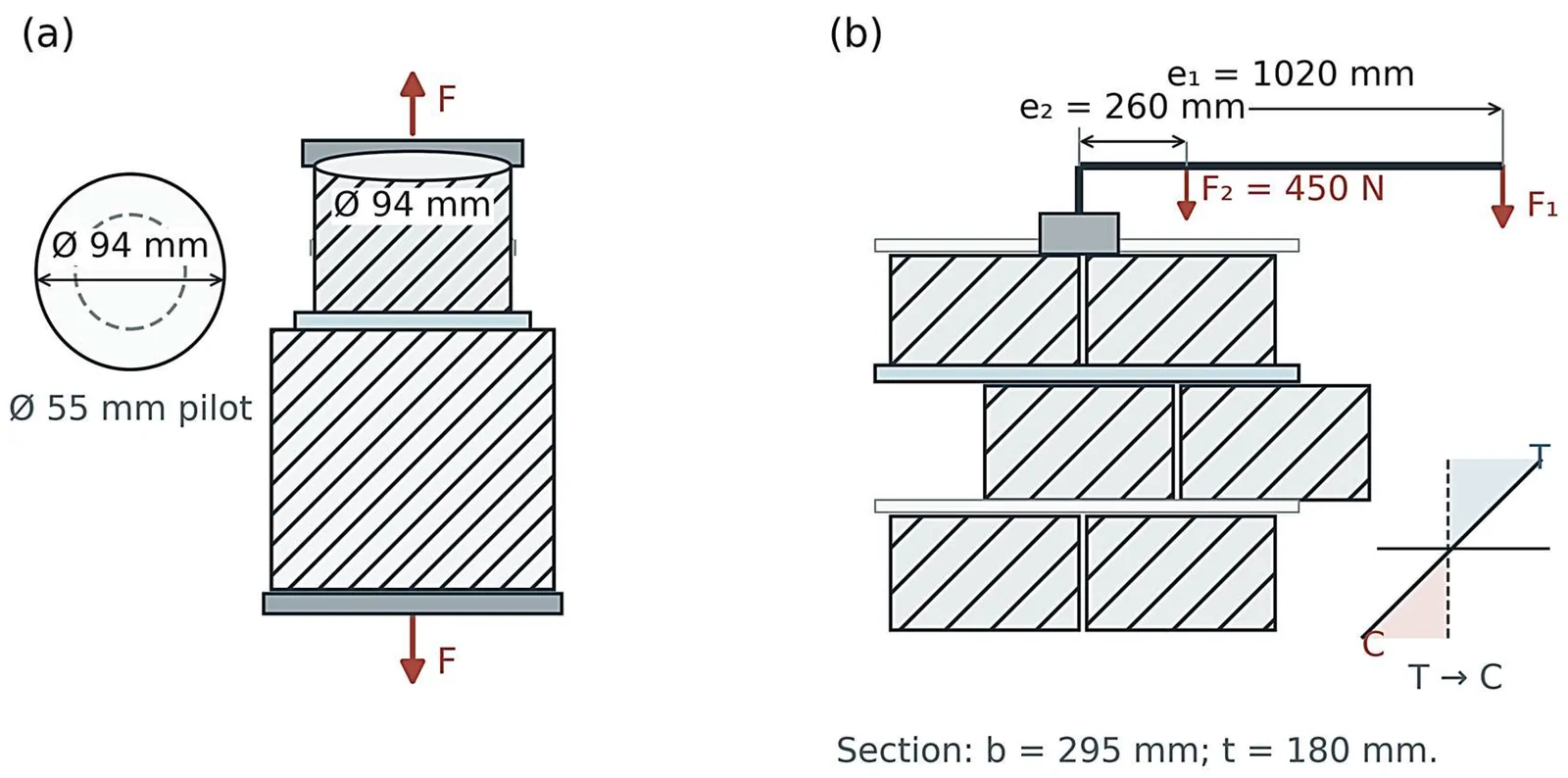
Test geometries and idealized load transfer: (**a**) direct axial tension on a 94 mm AAC core, with the 55 mm pilot area shown for scale comparison; and (**b**) lever-induced bond-wrench bending and the resulting idealized linear stress gradient. The 1 mm bed joint is exaggerated for visibility; drawings are not to scale. The stress fields are explanatory models, not measured maps. In (**a**), red arrows show the axial force F; in (**b**), they show F1 and F2, with e1 and e2 denoting lever arms. Hatched shapes represent AAC blocks and the light-blue strip the mortar joint. T and C mark tension and compression; the dashed line is the neutral axis, the sloping line the idealized stress gradient, and blue and pink regions indicate tension and compression, respectively.

**Figure 2:**
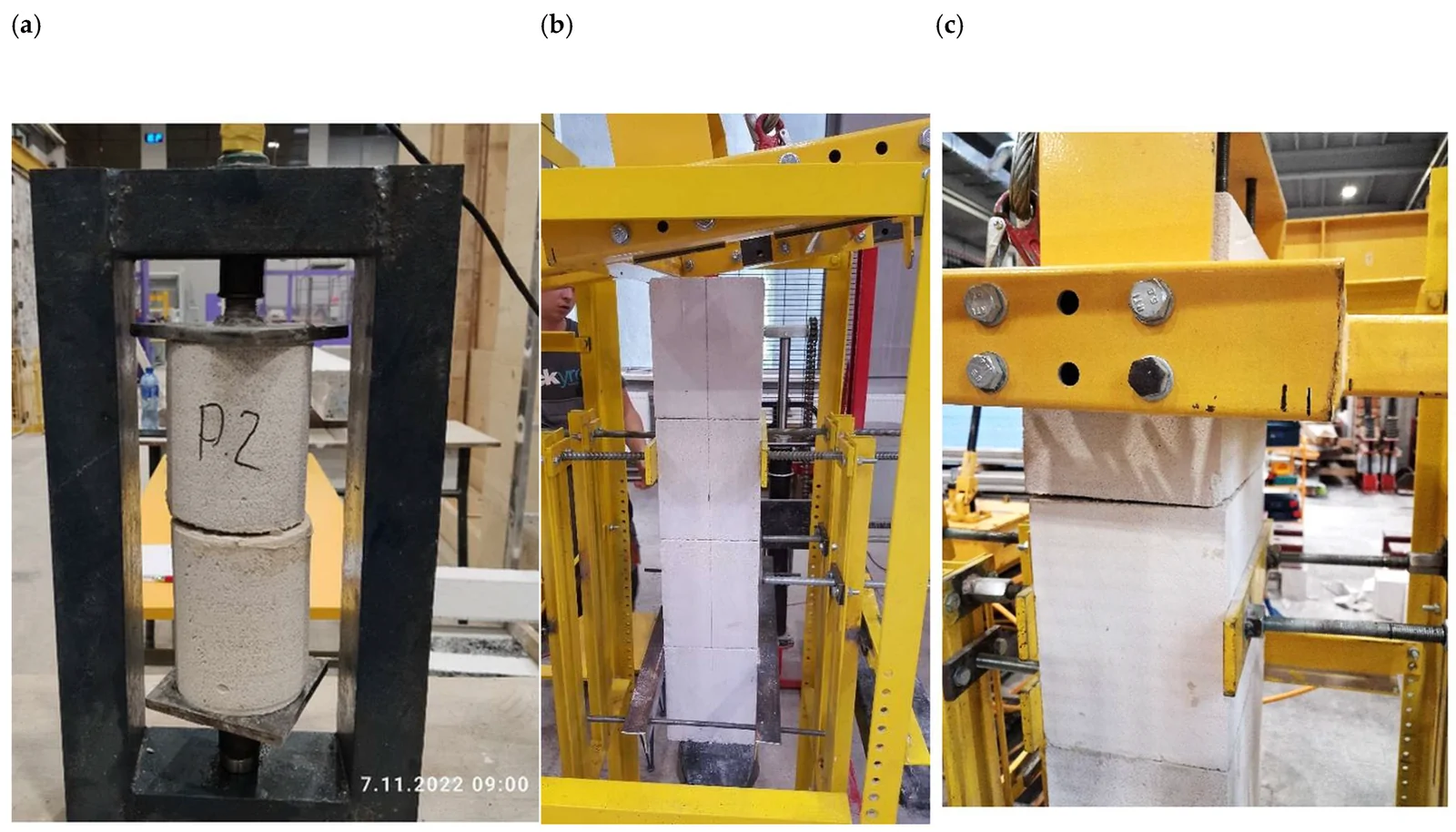
Representative photographs of the test setups: (**a**) 94 mm core in the axial test frame; (**b**) AAC assembly mounted for bond-wrench testing; and (**c**) joint region after wrench loading.

**Figure 3:**
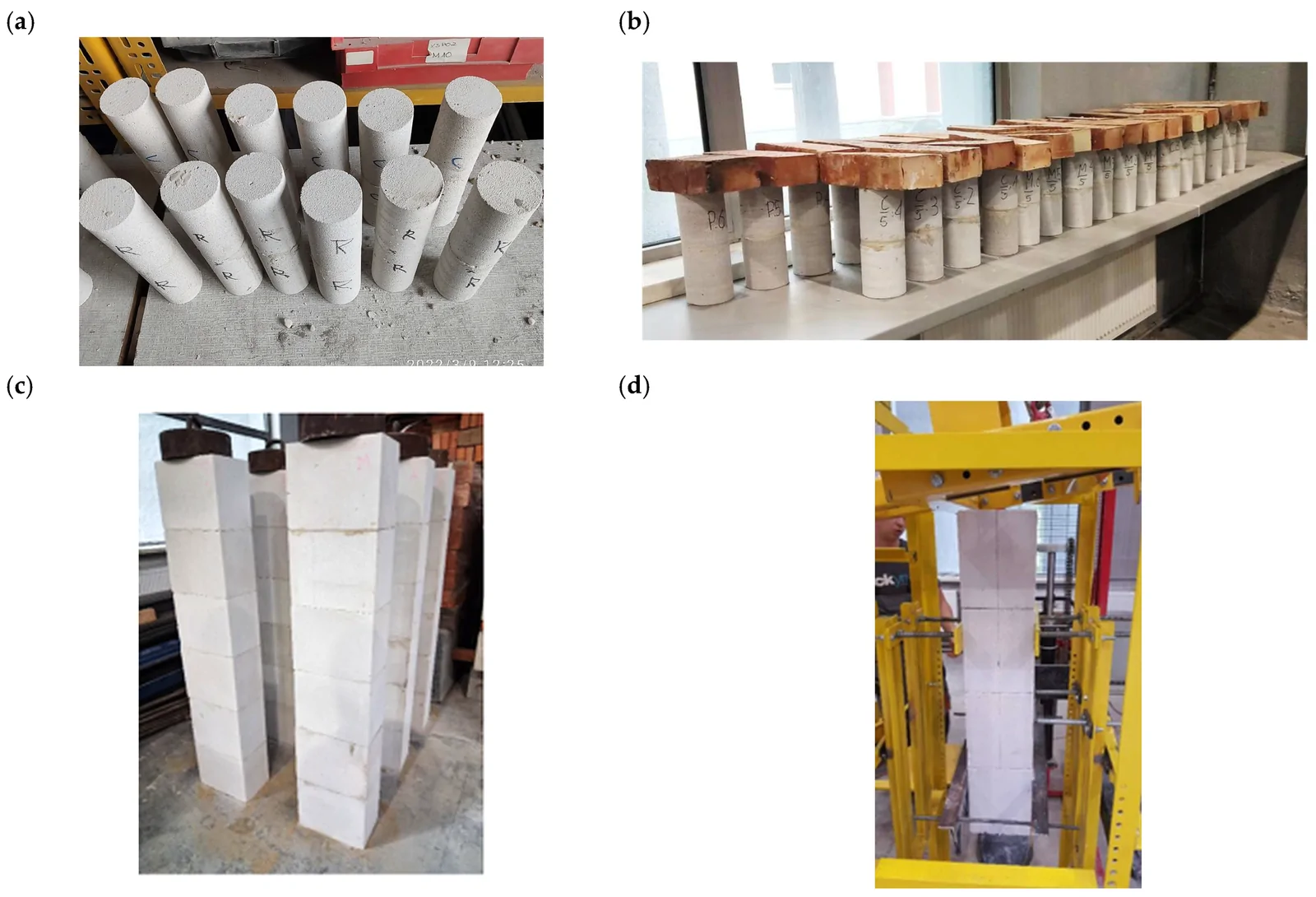
Specimen configurations from the experimental program: (**a**) bonded 55 mm AAC core specimens; (**b**) 94 mm core assemblies; (**c**) AAC masonry elements prepared for the bond-wrench series; and (**d**) a masonry assembly mounted in the wrench-test frame. The photographs document the configurations; they are not dimensional records.

**Figure 4:**
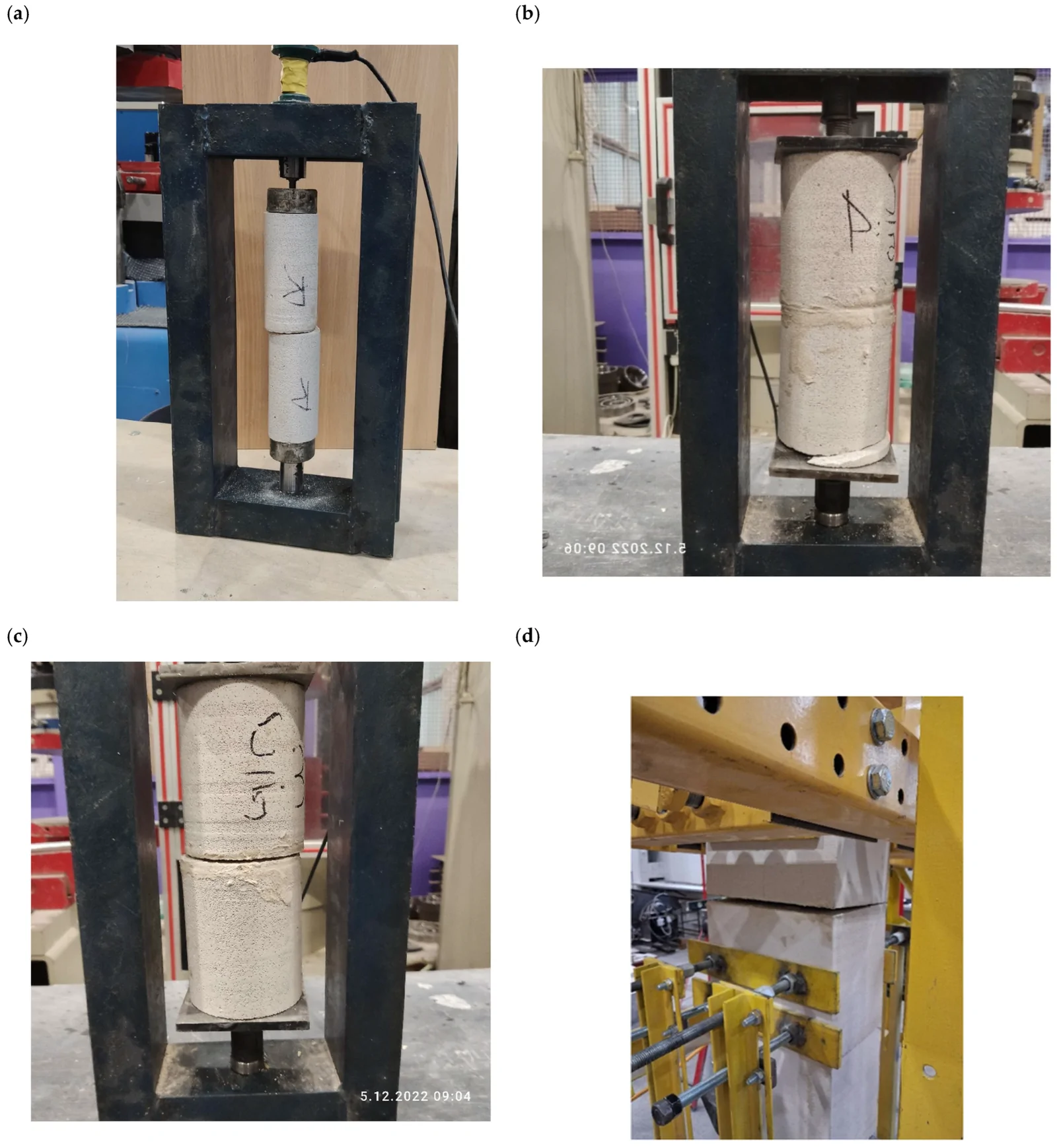
Representative post-test appearances: (**a**) a 55 mm pilot core specimen; (**b**) visible cracking in a 94 mm core away from an intended interface; (**c**) a 94 mm specimen with visible separation in the joint region; and (**d**) a masonry specimen after wrench loading. Photographs are illustrative and do not replace specimen-level failure classifications.

The specimen-level axial and wrench observations, means, standard deviations, and coefficients of variation (CV) were taken directly from the experimental dataset. Relative differences were calculated descriptively from group means. Formulation-mean ordinary least-squares (OLS) models were calculated without weighting for density versus compressive strength and compressive strength versus each bond metric; their unit of analysis is the formulation mean (n = 5 or 6), not the individual specimen. Leave-one-formulation-out slope ranges were calculated as a sensitivity check. Between-mixture analysis of variance (ANOVA) and post hoc comparisons were not recalculated; the wrench series has one assembly per mixture, and the remaining group sizes are small; thus, all formulation comparisons are presented descriptively. A post hoc single-outlier Dixon Q10 screen was applied within each 94 mm axial-core series, the complete 55 mm pilot series with at least three readings, and each recorded hardened-mortar density, flexural-strength, and compressive-strength replicate series (α = 0.05) [23,24]. No value was removed solely for being extreme. The five wrench readings per mixture were not subjected to an outlier test because they are successive joints from one assembly, not independent replicates. With only three to six values per series, normality could not be assessed reliably; because the screen was applied across multiple series without familywise adjustment, its results are exploratory. The force paths and idealized stress fields are summarized in Figure 1; representative test photographs, specimen configurations, and post-test appearances are shown in Figure 2, Figure 3 and Figure 4, respectively.

The reanalysis was performed from recorded force, area, and stress values rather than by reconstructing unreported load histories. Axial nominal stress was checked against the recorded bonded area, and wrench values were retained as tabulated because the full intermediate calculation precision was unavailable. No test-level *p*-values, confidence intervals, or significance claims are added.

## 3. Results

### 3.1. Preliminary 55 mm Core Series

The 55 mm series was not carried forward as a comparative strength dataset. The complete five-reading series for R, C.0, and P had no value flagged by the exploratory Dixon Q10 screen at α = 0.05; the M.0 and A series each contain only two available values and were not tested. One C.0 pilot row is internally inconsistent: specimen C.0.55.1 is reported as 24 N and 0.086 MPa, whereas 24 N over the nominal 2376 mm^2^ area corresponds to approximately 0.010 MPa. Both recorded values are reproduced in Supplementary Table S3, without correction. The available results present CV values above 56% for R, C.0, and P; M.0 and A specimens were damaged before testing or failed in an unintended manner. The later 94 mm bonded area is 2.92 times larger than the 55 mm area. A larger area may sample more of the heterogeneous AAC–mortar interface, but the study did not isolate diameter as the only changed variable; the higher scatter cannot be attributed to area alone. The 55 mm observations are, therefore, presented only as a preliminary test-development result. All available pilot force and stress observations are reproduced in Supplementary Table S3.

### 3.2. Axial Tensile Bond Strength

The 94 mm series yielded five valid interface failures for R, six for C.0, four for C, six for M, four for P, and four for A (Table 2). The reference mean was 0.378 MPa (SD 0.0648 MPa). C at 0.229 wt.% had the highest mean, 0.528 ± 0.0807 MPa, a descriptive increase of 39.7% over R. M and P averaged 0.444 and 0.470 MPa, corresponding to descriptive increases of 17.5% and 24.3%. Individual force and stress pairs are provided in Supplementary Table S1.

**Figure 5:**
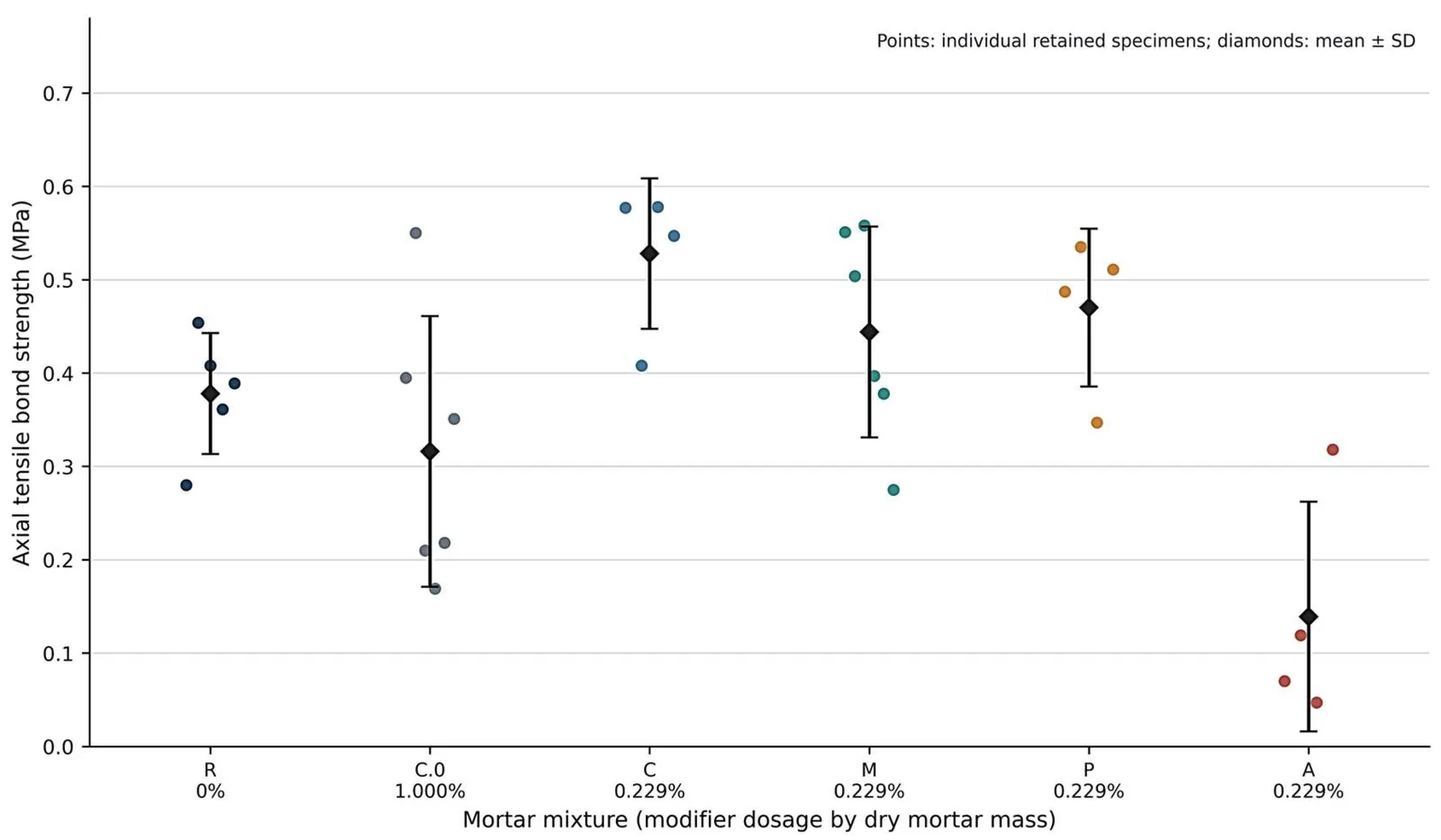
Individual valid axial tensile bond strengths for 94 mm AAC core specimens. Points show specimen values; black diamonds and bars show the mean and SD. Sample counts are listed in Table 2.

C.0 at 1.000 wt.% averaged 0.316 MPa with CV 45.8%, whereas A at 0.229 wt.% averaged 0.139 MPa with CV 89.1%. The A estimate is especially unstable relative to its mean. No 94 mm axial observation was flagged by the exploratory Dixon Q10 screen. The lowest C-series observation, C.94.2 (0.408 MPa), was closest to the threshold (Q = 0.818; Qcrit = 0.829 for n = 4) and was retained. All valid interface observations are retained in Table 2, including groups omitted from some earlier summary comparisons. Their scatter and the documented exclusions constrain interpretation; no claim of a confirmed optimum or statistically established modifier effect is made. Valid n counts reported interface failures. SD, standard deviation; CV, coefficient of variation. C.0 and A are retained despite high CV to show all reported valid interface results. The individual valid axial observations are plotted in Figure 5.

### 3.3. Bond-Wrench Flexural Strength

**Figure 6:**
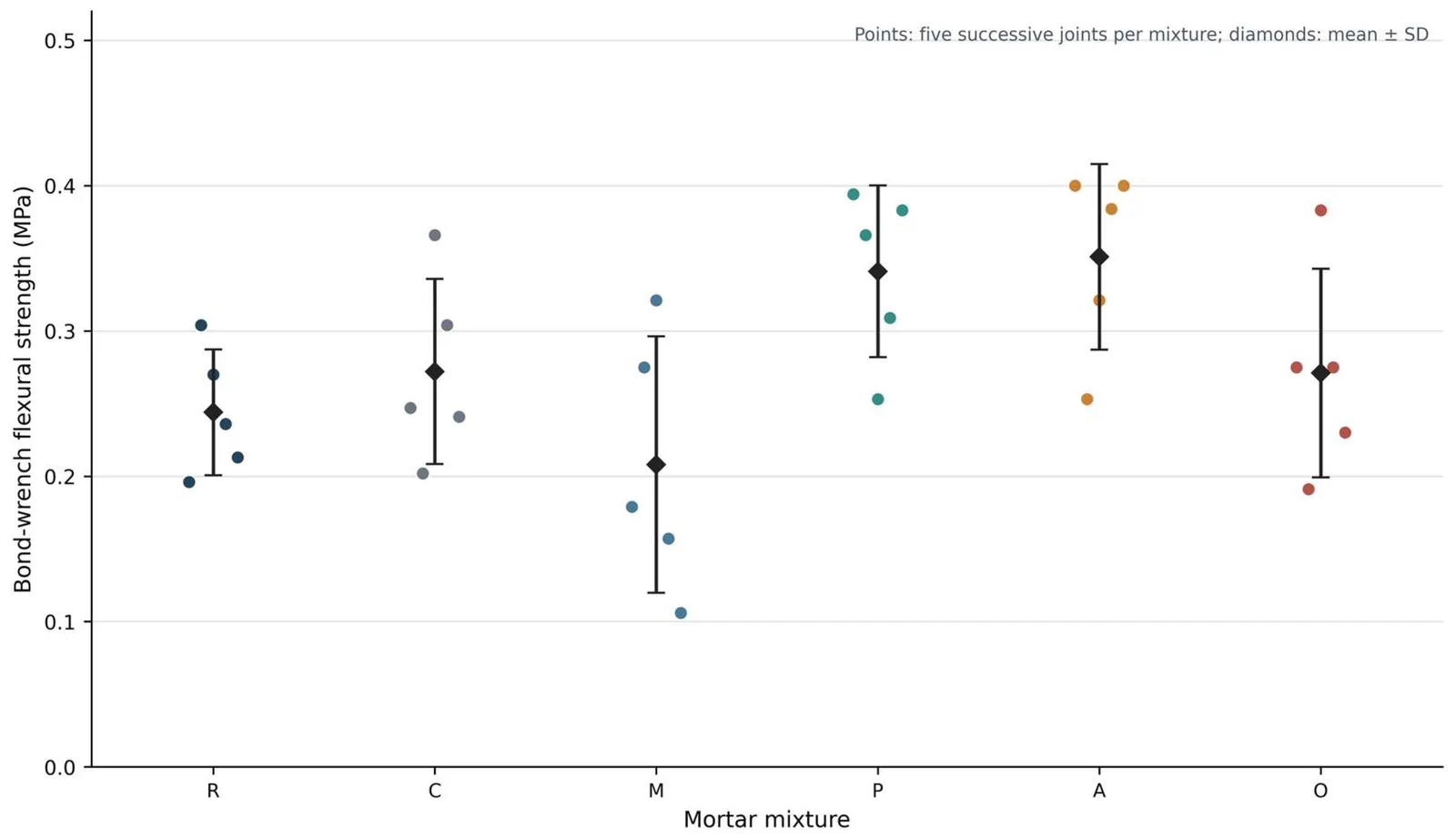
Reported joint-level bond-wrench edge stresses. Points represent five successive joints detached from one masonry assembly per mixture; black diamonds and bars show the mean and SD. The points are descriptive repeated observations, not independent replicates.

All reported bond-wrench specimens failed at the mortar–AAC interface. The reference mean was 0.244 MPa. P and A had the highest reported means, 0.341 and 0.351 MPa, approximately 39.8% and 43.9% above R. C and O were close at 0.272 and 0.271 MPa, while M had the lowest mean at 0.208 MPa. The M and O CV values were 42.5% and 26.5%. These group summaries are descriptive: each mixture was tested on one assembly, with five successive joint readings (Table 3). The five individual force, detached-weight, and calculated edge-stress records per mixture appear in Supplementary Table S2. The individual successive joint readings are plotted in Figure 6.

For each mixture, the five joint readings were taken successively from one masonry assembly as the blocks were detached. They are not five independent replicates, and this design cannot estimate between-assembly variability. No inferential comparison or statistical outlier test was applied to the wrench readings.

### 3.4. Density and Strength of Hardened Mortar

The tabulated mean density of each modified mortar was below the reference value of 1736 kg/m^3^. The largest reduction occurred for P (1264 kg/m^3^). Each modified mortar also had a lower tabulated compressive strength than R (7.05 MPa); M was lowest at 2.88 MPa. Flexural strength exceeded the tabulated reference value only for C (2.09 MPa) and O (1.83 MPa). The A.3 density reading (1578 kg/m^3^) was flagged as a potential high outlier by the exploratory Dixon Q10 screen (Q = 0.984; Qcrit = 0.970 for n = 3; α = 0.05). It was retained because the available records do not establish a measurement error; the Table 4 mean (1457 ± 105 kg/m^3^) and full-data models include it. The outlier flag is exploratory because n = 3 is too small for a reliable normality check. The individual density, flexural-strength, and compressive-strength readings are provided in Supplementary Tables S4–S6.

### 3.5. Descriptive Formulation-Mean Comparisons and Models

**Figure 7:**
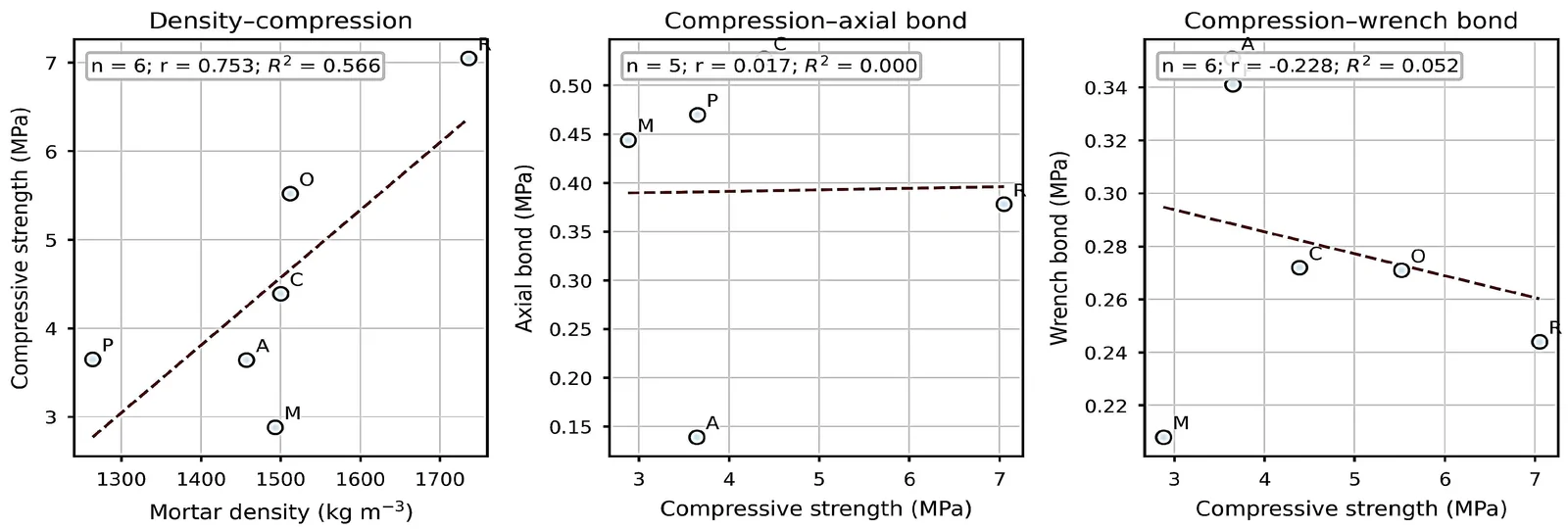
Exploratory unweighted linear fits to formulation means. Open circles mark formulation means; dashed lines show the fitted unweighted OLS trends. Small n and formulation-level aggregation preclude treating these regressions as specimen-level constitutive relationships.

Table 5 summarizes the arithmetic differences and relative changes in axial group means versus R. These comparisons are descriptive and are not significance tests. Table 6 and Figure 7 summarize the unweighted OLS models fitted to formulation means.

## 4. Analysis of Results and Discussion

### 4.1. Prior Evidence and the Specific Contribution

AAC masonry bonds have been investigated at both material and assembly scales. Jasiński and Galman reported tests on joints between AAC wall panels, while related work modelled and tested shear capacity in traditional AAC wall joints [25,26,27]. Silva and co-authors examined the interface between AAC masonry and reinforced concrete after compression [28]. These studies confirm the structural importance of interfaces in AAC construction; however, their wall-scale geometry, combined loading, or dissimilar substrate is not equivalent to the present direct tensile core test. Their results support the relevance of the topic, not a numerical benchmark for the present 0.1–0.5 MPa values.

A closer methodological comparison is provided by flexural bond-wrench studies on brick or clinker masonry. Gaggero and Esposito examined bond-wrench response and variability in brick masonry; Kubica and Galman studied mortar strength under different water contents and curing times; Galman and colleagues related wrench-test bond to water content and age [29,30,31]. These investigations make two points relevant here: masonry bond tests can show substantial variability, and moisture history or curing age can alter the measured response. The substrates and mortar systems differ; thus, the direction and magnitude of their effects cannot be transferred directly to AAC with a 1 mm white-cement joint.

Published AAC bond studies include direct comparisons of joint materials and test geometries. Raj et al. compared ordinary sand–cement and polymer-modified mortars and reported increased bond when an AAC interface was treated with cement slurry; Tandon et al. used cross-couplet tension and triplet shear tests across mortar mixtures and joint thicknesses [2,3]. Pure-tension and shear tests have also been applied to masonry–sprayed-mortar interfaces with different substrates and specimen geometries [32]. These studies establish that AAC bond research is well developed and that substrate treatment, mortar type, and test configuration matter; their results are not direct numerical benchmarks for the present 1 mm white-cement joint.

Within the literature located for this revision, we did not identify an earlier study combining axial tension of 94 mm cores spanning an AAC bed joint measured at 1 mm, EN 1052-5 bond-wrench testing of masonry assemblies, and the present coded low-dose admixture screen. The contribution is this bounded test configuration and paired-method comparison. We do not claim to introduce tensile bond measurement, cored masonry testing generally, or AAC bond research.

### 4.2. Stress Distribution, Bonded Area, and the 1 mm Joint

The axial core test is interpreted through a nominal mean stress F/A_β_, assuming centered loading and uniform stress over the circular bonded plane. This assumption is sensitive to eccentricity. For a circular section under an axial force F with eccentricity e, elementary elastic-section theory obtains σmax/σnom = 1 + 8e/d. Thus, a hypothetical 1 mm eccentricity corresponds to an 8.5% edge-stress increase at d = 94 mm and 14.5% at d = 55 mm. These are model sensitivities, not measured corrections; alignment was not recorded. The 94 mm bonded area (6940 mm^2^) samples almost three times the area of the preliminary 55 mm configuration (2376 mm^2^), which can reduce dependence on a single local pore, surface defect, or incomplete-contact patch. It does not eliminate AAC heterogeneity, nor does the observed pilot scatter prove that area alone caused the improvement.

The area ratio also places the 55 mm pilot in perspective. The bonded planes are 2376 mm^2^ and 6940 mm^2^; thus, the 94 mm plane samples approximately 2.92 times the area. If local defects are spatially distributed, a larger area may average over more pores and surface irregularities; however, the larger core also changes the probability of edge effects, specimen damage, and non-uniform load transfer. Because diameter was changed together with specimen preparation and test-development decisions, the area ratio cannot be interpreted as an isolated size-effect experiment. The pilot’s coefficient of variation above 56% for several mixtures and the small number of valid pilot failures are reasons to exclude it from the main mixture ranking, not proof that a larger area alone improves precision.

The core procedure is distinct from the conventional EN 1542 pull-off test, which uses a localized bonded loading fixture and applies to products on concrete rather than this AAC masonry system [18]. The pull-off literature warns that a small local test region can be affected by substrate heterogeneity, eccentricity, and damage introduced during coring; failure outside the target interface may also limit what the test measures [19,20]. The 55 mm core pilot is not presented as a standard pull-off result; it was a direct axial core test with high scatter and invalid failure modes. The adopted 94 mm area improves spatial representation, but only a controlled diameter study with documented alignment and failure surfaces could quantify the size effect.

The issue of the sampled area is a known concern in the pull-off testing of non-homogeneous substrates: a small test footprint may represent a local weak zone or unusually dense patch, while the annular cutting operation can alter the surrounding material. A result can also be controlled by tensile failure within the substrate rather than by the interface of interest [18,19,20]. This motivated the larger axial core configuration in the present program. The current method still has its own limitations: the steel plate and adhesive create an indirect force path, manual screw loading may introduce variable rate, and unmeasured eccentricity can produce a non-uniform stress field. The calculated expression σ_max_/σ_nom_ = 1 + 8e/d illustrates the sensitivity of a circular section to eccentricity: for a hypothetical 1 mm offset, σ_max_ is about 8.5% above nominal at 94 mm and 14.5% above nominal at 55 mm. These are ideal elastic sensitivities, not corrections to the results, because no eccentricity was measured.

### 4.3. Modifier Dosage, Fresh Consistency, and Strength Development

A 0.229 wt.% addition is only 2.29 g per kg of dry mortar, or 57.25 g per 25 kg batch. This small mass may appear economical; however, cost benefit cannot be determined without price and active-solids data. Small doses can alter yield stress, plastic viscosity, water retention, and contact formation. The available test notes describe poor workability at 1.000 wt.% for C.0 and M.0; however, fresh consistency and its retention over time were not measured. A 2024 AAC thin-bed mortar study reported improved workability and slower hardening at a 15% PVAc addition [12]; its different dose and formulation do not establish a bond response for the low-dose coded modifiers here. This omission matters especially for a joint measured at 1 mm, in which small changes in spread and water demand can affect coverage. Published polymer-mortar studies also show formulation-dependent rheological and strength responses [8,9].

The public product profile describes a dry blend of hydraulic binders, selected mineral aggregates, and admixtures, and specifies a maximum aggregate size below 0.8 mm [14]. It does not disclose a complete formulation or verify the tested batch. The joint was measured at 1 mm, but actual aggregate grading and individual joint locations were not measured; thus, within-joint thickness variation cannot be quantified. Particle packing and mortar coverage may, therefore, be sensitive to the thin joint; the available data do not show grain bridging, incomplete contact, or a cause of any failure. An addition of 0.229 wt.% relative to dry mortar is 2.29 g/kg; without a verified cement fraction it cannot be expressed as a cement-mass percentage. Cellulose-ether studies report changes in water retention, viscosity, and consistency [33,34,35,36,37]. In a 1 mm joint, these properties could affect spreading and contact with suction-active AAC. Flow, open time, water retention, and fresh-air content were not measured. Total mixing water was fixed at 5.75 L per 25 kg and included water carried by liquid O; however, O’s water contribution was not quantified separately.

The A-coded mix requires particular caution. The descriptor “vinyl alcohol” does not verify PVA chemistry. Its tabulated flexural and compressive strengths (1.17 and 3.64 MPa) were below the reference values (1.77 and 7.05 MPa), and its axial bond mean was low, while its bond-wrench mean was comparatively high. The available data do not identify the additive sufficiently to explain these different results. No setting times, hydration kinetics, exact test age, or strength-development curve were recorded. The literature on polymer-modified mortars reports age- and formulation-dependent strength changes [8,10]; however, those results do not establish a mechanism for the present coded mixture.

Studies of polyvinyl alcohol-modified Portland-cement mortars and calcium–sulfoaluminate repair mortars report formulation-dependent rheological and mechanical responses [7,8,9,10]. These systems provide context for why additive identity and test age matter, but they cannot explain the A-coded results here. The recorded descriptor “vinyl alcohol” does not confirm PVA identity; the binder formulation is also incomplete, and the study did not measure setting time, calorimetry, hydration kinetics, or strength development over time. These literature findings, therefore, motivate future testing but do not provide a causal explanation of the current measurements.

The 1.000 wt.% C.0 and M.0 mixtures were described as too dense and poorly workable after the preliminary series; therefore, their dosages were reduced. This is a qualitative observation and does not establish a dose–response relationship for any modifier. Published dry-mix mortar research has examined effects of chemical admixtures on working and mechanical properties [38], but fresh-state variables were not measured here. Flow-table spread, plunger penetration, correction time, and retention after a specified interval were not recorded. Such measurements, alongside a controlled water series, would be needed to distinguish changes in fresh-state behavior from other material responses.

### 4.4. Candidate Chemistry-Dependent Pathways and Compositional Limits

The chemical interpretation of the bond results is constrained by incomplete identification of the coded modifiers. The available descriptions identify M as methylcellulose; “M-polymer acrylic”, “vinyl alcohol”, and “vinyl acetate” are broad labels that do not establish the exact composition, polymer grade, active-solids content, or carrier of P, A, and O. The C-coded powder’s public product card describes a re-dispersible synthetic-resin powder but does not disclose its precise polymer family [16]. Public mortar product records provide context for the base mortar, not a verified composition for the tested batch. The chemical pathways discussed below are, therefore, hypotheses, not causal explanations of the observed rankings.

The modifier log identifies M as methylcellulose, but its grade, molecular mass, and substitution level are unknown. Cellulose ethers can change water retention, consistency, pore–solution viscosity, and early cement hydration [6,33,34,35,36,38,39,40,41]. A 2023 tile-adhesive study linked hydroxyalkyl methylcellulose characteristics to early skin formation, while a 2025 review summarizes how cellulose-ether chemistry and molecular structure affect water retention and fresh-state properties [42,43]. These findings make a change in wetting or hydration plausible; however, they cannot establish the mechanism for M in the present joint. No flow, water-retention, hydration, calorimetry, pore-structure, or interface-chemistry measurements were made here.

Polymer chemistry can change tensile adhesion in cementitious systems; however, the response depends on formulation, dosage, and test method. Kulesza et al. tested ten re-dispersible polymer powders at 1–5% of mortar mass with mixing water fixed at 20% of mortar mass. At the 5% dosage of redispersible polymer powder (RDP), the published Table 4 presents tensile adhesion of 2.61 MPa for the vinyl-acetate homopolymer H5 and 0.28 MPa for the unmodified reference; the acrylic homopolymer A0 reached 0.65 MPa. Table 4 lists 2.29 MPa for styrene–acrylic S0, 2.13 MPa for S5, and 2.23 MPa for vinyl-acetate–ethylene E11; the accompanying narrative reverses the last two values [44]. H5 provided the highest tensile-adhesion value within that study. Its 5% dosage is far above the present C dose of 0.229 wt.%, and its mortar/test system differs from a 1 mm AAC masonry joint; these numbers are a context, not a direct benchmark. Lee’s 2024 acrylic-ester study reported a 35% smaller average particle size for one modified polymerization route and 78–113% bond-strength gains across its polymer-modified mortars; the bond test used a 5 mm layer on concrete [45]. Ma et al. studied ethylene-vinyl acetate (EVA)-modified repair mortar under underwater bonding conditions [46]; this is a different polymer family and service environment. These studies support formulation-specific effects, not an identity or effect estimate for coded C or O. O was a liquid addition at 0.687 wt.% of dry mortar, but its polymer form, solids, and carrier are unknown. The label “vinyl acetate” alone does not establish that O is PVAc, H5, or EVA. C had the highest axial-core mean, whereas P and A had the highest bond-wrench means; comparisons across these tests or dissimilar methods in the literature cannot establish a general chemical ranking.

**Figure 8:**
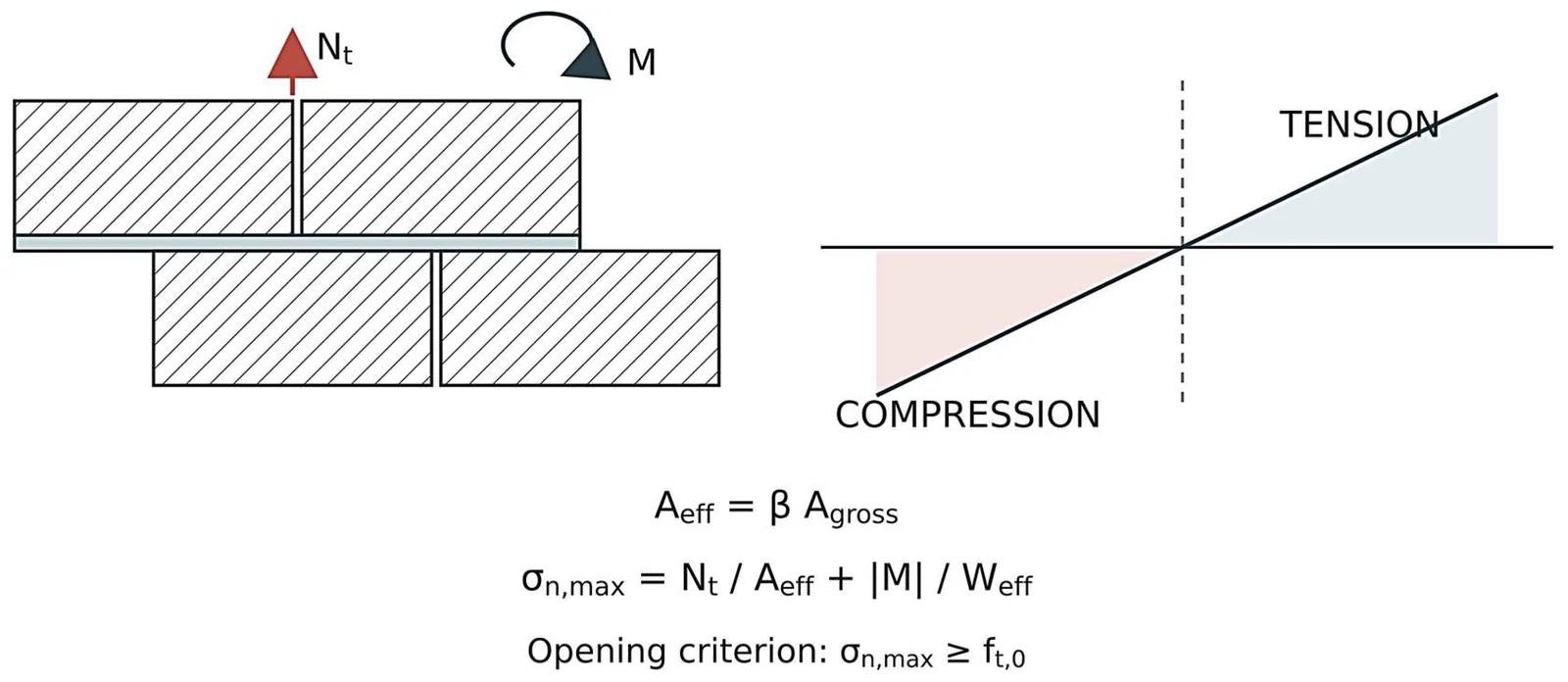
Schematic masonry joint and idealized section-stress model. The nominal 1 mm bed joint is exaggerated for clarity; its final thickness was not measured after assembly. The equations show an assumed effective contact area and opening criterion under axial force and bending moment; the stress gradient is conceptual, not a measured stress field. Red arrow N_t denotes axial tension and curved arrow M the bending moment. Blue and pink areas mark tension and compression; the dashed line is the neutral axis and the diagonal line the idealized stress gradient. A_eff is the effective contact area, β the assumed contact fraction, A_gross the gross area, σ_n,max the maximum normal stress, W_eff the effective section modulus, and f_t,0 the assumed tensile threshold in the opening criterion.

Taken together, the literature identifies hydration timing, polymer-film formation, water retention, and surface coverage as pathways worth testing, but it cannot explain the present ranking retrospectively. P, A, O, and C are not fully characterized; active-solids content, the separate amount of water carried by O, flow, fresh air, contact area, and hydration timing were not measured. The total mixing-water dose was 5.75 L per 25 kg and included O water. The measured result is method-dependent: C leads in core-axial tension, whereas P and A lead in the bond-wrench test. Figure 8 separates the public product-profile context for the base mortar from candidate hydration and contact pathways and the bed-joint response model; dashed links are hypotheses, not measured effects. The data do not establish a general chemical ranking or a molecular mechanism for these differences [32,42,43,44,45,46].

### 4.5. Formulation-Mean Models and Limits of Inference

The six-formulation means show a positive density–compressive-strength association (r = 0.753, R^2^ = 0.566); however, leave-one-formulation-out slopes vary substantially. In a sensitivity calculation, replacing the A density mean with the mean of the two remaining values after excluding A.3 (1396.5 kg/m^3^) changed the six-formulation slope only slightly (0.00763 to 0.00760 MPa/(kg m^−3^)); r changed from 0.753 to 0.774 and R^2^ from 0.566 to 0.599. The full-data model remains primary. In contrast, compressive strength was not linearly associated with the axial bond across the five shared formulations (r = 0.017, R^2^ < 0.001) or with the wrench bond across six formulations (r = −0.228, R^2^ = 0.052). These are unweighted ecological regressions of group means, not specimen-level material laws. They show that mortar-prism compression alone did not reproduce the method-specific bond ranking in this small dataset; they do not establish absence of a physical relation at the specimen or interface scale. The different rankings visible in Figure 5 and Figure 6 are consistent with different mechanics: mean axial stress over a circular plane versus edge tensile stress from lever-induced bending. These models are based on six or fewer points and describe the six observed mixtures; they are not forecasts for new mixtures.

The density–compression fit has a positive slope of 0.00763 MPa per kg/m^3^ and an R^2^ of 0.566. Its leave-one-formulation-out slope ranges from 0.00301 to 0.01250 MPa per kg/m^3^, showing that the estimated gradient depends on which mixture is omitted. By comparison, the axial-bond regression against compression has a slope near zero (0.00155 MPa/MPa; R^2^ = 0.0003), while the wrench regression has a small negative slope (−0.00829 MPa/MPa; R^2^ = 0.052). Their leave-one-out slope ranges include both negative and positive values. These models, therefore, do not support a stable linear relation between companion prism compression and either bond metric in this dataset.

The descriptive mean differences in Table 5 rest on small groups; the high scatter in A and exclusions of unintended failures further limit stable effect estimation. The wrench design used one masonry assembly per mixture, with five sequential joint readings; it cannot estimate between-assembly variability. Confirmation requires several independently constructed assemblies per mix, prespecified failure criteria, raw force and area records, verified modifier identity and active solids, a quantified contribution of O to total water, measured fresh consistency and retention, substrate moisture and surface condition, measured final joint thickness, curing age and temperature–humidity values, and documented alignment and loading rate.

A targeted follow-up program should combine independent masonry assemblies for every formulation with a measured joint-thickness distribution centered on 1 mm, fixed total mixing-water dosage, verified additive identity and solids contents, and replicate measurements of fresh consistency and its time dependence. For any liquid modifier, the amount of product water included in the total should be quantified. EN 1015-3 provides a flow-table approach to consistency, EN 1015-9 addresses workable life and correction time for thin-layer mortars, and EN 1015-7 can quantify fresh air content [47,48,49]. Characterizing AAC water absorption or capillary uptake according to EN 772-11 would help to control a key substrate variable [50]. The cited EN 196-1 mixing procedure should also be checked against the actual mortar batching protocol [22]. A future design should report test age, temperature and humidity, AAC moisture state, loading rate, alignment tolerance, individual failure surfaces, and the number of independently built assemblies; these controls would make the modifier comparison more reproducible.

These regressions are ecological summaries of mixture means. Their predictor values are not paired specimen-level measurements, the number of formulations is small, and the mixtures differ in unknown active-solids content and may have different water demand despite the fixed mixing-water dosage. They cannot test an interface-scale law or separate modifier effects from density, air content, hydration, and surface moisture. The density–compression trend is physically plausible as a broad mixture response; however, six points do not provide a general constitutive relation. The weak bond models, likewise, do not prove that mortar bulk strength is irrelevant: direct interfacial tensile strength depends on contact continuity, pore structure near the substrate, and local fracture path, none of which is represented by prism compression alone.

AAC tensile bond has previously been measured with cross-couplet specimens and direct-tension tests have been used for other masonry–mortar interfaces [2,3,32]. The additional 2023 study of PVAc-based thin-bed mortar for AAC [11] examined compressive and splitting-tensile response rather than direct tensile adhesion across the AAC–mortar interface. In the literature located for this revision, we did not identify a published study pairing 94 mm axial cores bonded across an AAC bed joint measured at 1 mm with EN 1052-5 bond-wrench tests on masonry assemblies and a low-dose modifier screen. The contribution is this specific test configuration and method-dependent comparison, not the first study of AAC bond.

## 5. Conclusions

The C mixture at 0.229 wt.% had the highest axial mean (0.528 MPa versus 0.378 MPa for R), while P and A had the highest bond-wrench means; A combined a low axial mean (0.139 MPa) with the highest wrench mean (0.351 MPa). The method-dependent ranking limits cross-comparison: axial tension and the bond-wrench test produced distinct responses in this dataset. The C-coded material is disclosed only as a re-dispersible synthetic-resin powder; M is labelled methylcellulose, while P, A, and O remain identified only by broad recorded descriptions. O was liquid, and its water was included in the 5.75 L per 25 kg total mixing-water dose; the product-water amount was not reported separately.

All modified mortars had lower tabulated density and compressive strength than the reference, although the tabulated and narrative reference-strength summaries conflict. The 0.229 wt.% dosage corresponds to only 2.29 g/kg of dry mortar, but neither economic benefit nor a fresh-consistency advantage can be established without composition, price, flow, and retention data. Available test notes describe the 1.000 wt.% C.0 and M.0 mixtures as poorly workable, underscoring that small dosage changes can matter in a 1 mm joint.

The public profile for a matching M5, Type T, white-cement AAC thin-bed mortar specifies 5.75 L water per 25 kg, aggregate size below 0.8 mm, and a maximum 3 mm bed joint [14]. It does not identify the tested production batch. All study formulations used 5.75 L of total mixing water per 25 kg, including water carried by O; the O contribution was not quantified separately. The 94 mm core series sampled more of the bonded area than the highly scattered 55 mm pilot; however, the area effect was not isolated. Confirmation requires verified additive chemistry, independent wrench assemblies, controlled fresh-state properties, and a documented, location-specific joint-thickness distribution with the measurement method reported, alongside curing, alignment, and failure-mode records.

## Figures and Tables

**Table 1:** Anonymized modifier descriptions, available composition, mass dosage, and equivalent addition amounts.

Code	Recorded Label and Form	Dose (wt.% Dry Mix; g/kg; g/25 kg)	Available Composition/Unresolved Details
R	No modifier; reference	0; 0; 0	Reference mortar
C.0	C-coded re-dispersible synthetic-resin powder	1.000; 10.00; 250.0	Synthetic-resin-based re-dispersible powder; exact polymer family, auxiliaries, and active-solids content not disclosed [17]
C	C-coded re-dispersible synthetic-resin powder	0.229; 2.29; 57.25	Synthetic-resin-based re-dispersible powder; exact polymer family, auxiliaries, and active-solids content not disclosed [17]
M.0	Methylcellulose; powder	1.000; 10.00; 250.0	Cellulose ether; grade, molecular mass, substitution, and active-solids content unknown
M	Methylcellulose; powder	0.229; 2.29; 57.25	Cellulose ether; grade, molecular mass, substitution, and active-solids content unknown
P	“M-polymer acrylic”; powder	0.229; 2.29; 57.25	Acrylic-based family per recorded description; exact polymer type and active-solids content unknown
A	“Vinyl alcohol”; powder	0.229; 2.29; 57.25	Recorded descriptor does not verify PVA identity, grade, or active-solids content
O	“Vinyl acetate”; liquid	0.687; 6.87; 171.75	Polymer form, solids, and carrier unknown; water carried by O was included in total mixing water

**Table 2:** Axial tensile bond strength from the 94 mm core series.

Mix	Modifier Dosage	Valid n	Mean ± SD (MPa)	CV (%)
R	None	5	0.378 ± 0.0648	17.1
C.0	1.000 wt.%	6	0.316 ± 0.145	45.8
C	0.229 wt.%	4	0.528 ± 0.0807	15.3
M	0.229 wt.%	6	0.444 ± 0.113	25.4
P	0.229 wt.%	4	0.470 ± 0.0846	18.0
A	0.229 wt.%	4	0.139 ± 0.123	89.1

**Table 3:** Bond-wrench edge tensile stress by mortar mixture.

Mix	Modifier Dosage	Joints Reported	Mean ± SD (MPa)	CV (%)
R	None	5	0.244 ± 0.0433	17.8
C	0.229 wt.%	5	0.272 ± 0.0637	23.4
M	0.229 wt.%	5	0.208 ± 0.0882	42.5
P	0.229 wt.%	5	0.341 ± 0.0591	17.3
A	0.229 wt.%	5	0.351 ± 0.0639	18.2
O	0.687 wt.%	5	0.271 ± 0.0718	26.5

Five joint-level measurements per mixture came from one assembly and were obtained sequentially. They are repeated observations within that assembly, not independent replicates. SD, standard deviation; CV, coefficient of variation.

**Table 4:** Reported density and strength of hardened mortar specimens.

Mix	Density (kg/m^3^)	Flexural Strength (MPa)	Compressive Strength (MPa)
R	1736 ± 22.9	1.77 ± 0.568	7.05 ± 1.41
C	1500 ± 94.7	2.09 ± 0.423	4.39 ± 0.760
M	1493 ± 26.6	1.45 ± 0.306	2.88 ± 0.449
P	1264 ± 23.9	1.23 ± 0.166	3.65 ± 0.491
A	1457 ± 105	1.17 ± 0.288	3.64 ± 0.659
O	1512 ± 25.6	1.83 ± 0.267	5.52 ± 0.518

Values are mean ± SD as listed in the experimental dataset. Replicate counts and test age are not stated. A separate narrative summary presents reference flexural and compressive strengths of 2.22 and 6.50 MPa, which differs from the values in Table 4 (1.77 and 7.05 MPa).

**Table 5:** Descriptive axial tensile mean differences relative to reference mixture R.

Comparison	Δ Mean (MPa)	Relative Change
C.0 − R	−0.062	−16.4%
C − R	+0.150	+39.7%
M − R	+0.066	+17.5%
P − R	+0.092	+24.3%
A − R	−0.239	−63.2%

Δ is the arithmetic difference between group means; relative change is calculated against reference mean R. No confidence intervals or significance tests are presented.

**Table 6:** Unweighted OLS fits to formulation means and leave-one-formulation-out (LOO) slope sensitivity.

Response Model	n	Slope	r	R^2^	LOO Slope Range
Compression ~ density	6	0.00763 MPa/(kg m^−3^)	0.753	0.566	0.00301 to 0.01250 MPa/(kg m^−3^)
Axial bond ~ compression	5	0.00155 MPa/MPa	0.017	0.0003	−0.0202 to 0.0555 MPa/MPa
Wrench bond ~ compression	6	−0.00829 MPa/MPa	−0.228	0.052	−0.0286 to 0.00409 MPa/MPa

The predictors and responses are formulation-level means, not independent specimen measurements. LOO, leave-one-formulation-out; OLS, ordinary least squares. No *p*-values or causal interpretations are assigned.

## Data Availability

The original contributions presented in this study are included in the article and Supplementary Materials. Further inquiries can be directed to the corresponding author.

## Supplementary Materials

The following supporting information can be downloaded at: https://www.mdpi.com/article/10.3390/ma19214629/s1, Supplementary Tables S1–S6 present the specimen-level force, nominal stress, density, and strength readings used in the reanalysis. A dash indicates that no valid measurement was available. Table S1. Individual valid tensile results for 94-mm AAC cores; Table S2. Individual bond-wrench readings by mortar mixture; Table S3. Individual tensile results from the preliminary 55-mm core series; Table S4. Individual density measurements; Table S5. Individual flexural-strength measurements; Table S6. Individual compressive-strength measurements.

## Abbreviations

The following abbreviations are used in this manuscript:

AAC	autoclaved aerated concrete
CV	coefficient of variation
SD	standard deviation
LOO	leave-one-formulation-out
OLS	ordinary least squares
