# Supplementary material for: Method-Dependent Bond Strength of Thin-Bed Mortars with Coded Modifiers on Autoclaved Aerated Concrete
Source: Materials (Basel). 2026 Oct 31;19(21):4629. doi: 10.3390/ma19214629 (PMC13764904; doi:10.3390/ma19214629)
Supplement: Supplementary file 1 [file materials-19-04629-s001.zip › materials-4659609-supplementary.pdf]

Supplementary

Method-Dependent Bond Strength of Thin-Bed Mortars with Coded Modifiers on Autoclaved Aerated Concrete

Beata Łaźniewska-Piekarczyk [1,*] and Adam Piekarczyk [2]

1 Department of Building Processes and Building Physics, Faculty of Civil Engineering, Silesian University of Technology, Akademicka 5, 44-100 Gliwice, Poland
2 Department of Building Structures, Faculty of Civil Engineering, Silesian University of Technology, Akademicka 5, 44-100 Gliwice, Poland; adam.piekarczyk@polsl.pl
* Correspondence: beata.lazniewska@polsl.pl

Supplementary Table S1. Individual valid tensile results for 94-mm AAC cores.

Mix	Specimen ID	Maximum force (N)	Reported bond stress (MPa)
R	R.94.1	1941	0.280
R	R.94.2	3152	0.454
R	R.94.3	2834	0.408
R	R.94.4	2506	0.361
R	R.94.5	2700	0.389
C.0	C.0.94.1	2744	0.395
C.0	C.0.94.2	3820	0.550
C.0	C.0.94.3	1459	0.210
C.0	C.0.94.4	1174	0.169
C.0	C.0.94.5	1516	0.218
C.0	C.0.94.6	2436	0.351
C	C.94.1	4003	0.577
C	C.94.2	2834	0.408
C	C.94.3	4012	0.578
C	C.94.4	3795	0.547
M	M.94.1	3826	0.551
M	M.94.2	3495	0.504
M	M.94.3	3874	0.558
M	M.94.4	2757	0.397
M	M.94.5	2620	0.378
M	M.94.6	1907	0.275
P	P.94.1	3383	0.487
P	P.94.2	3711	0.535
P	P.94.3	2405	0.347
P	P.94.4	3549	0.511
A	A.94.1	486	0.0700
A	A.94.2	824	0.1190
A	A.94.3	327	0.0471
A	A.94.4	2208	0.3180

Table entries list the recorded force and stress values. "Valid" denotes the interface-failure series; the number of observations varies by mixture.

Supplementary Table S2. Individual bond-wrench readings by mortar mixture.

Mix	Joint ID	F_1 (N)	Detached-part weight g (N)	Edge stress (MPa)
R	R.W.1	280	78.60	0.196
R	R.W.2	470	77.94	0.304
R	R.W.3	410	78.44	0.270
R	R.W.4	350	76.89	0.236
R	R.W.5	310	77.03	0.213
C	C.W.1	370	77.81	0.247
C	C.W.2	290	77.32	0.202
C	C.W.3	580	78.44	0.366
C	C.W.4	470	78.76	0.304
C	C.W.5	360	79.08	0.241
M	M.W.1	250	77.55	0.179
M	M.W.2	420	79.59	0.275
M	M.W.3	500	77.40	0.321
M	M.W.4	210	76.58	0.157
M	M.W.5	120	78.72	0.106
P	P.W.1	630	78.60	0.394
P	P.W.2	580	79.97	0.366
P	P.W.3	380	78.20	0.253
P	P.W.4	480	77.79	0.309
P	P.W.5	610	79.82	0.383
A	A.W.1	640	77.08	0.400
A	A.W.2	380	78.26	0.253
A	A.W.3	500	79.31	0.321
A	A.W.4	610	78.54	0.384
A	A.W.5	640	79.47	0.400
O	O.W.1	420	80.06	0.275
O	O.W.2	270	77.81	0.191
O	O.W.3	610	80.77	0.383
O	O.W.4	420	82.28	0.275
O	O.W.5	340	78.35	0.230

Five successive joint readings per mixture came from one masonry assembly and were obtained during sequential block detachment. They are repeated observations within an assembly, not independent replicates.

Supplementary Table S3. Individual tensile results from the preliminary 55-mm core series.

Mix	Specimen ID	Maximum force (N)	Reported bond stress (MPa)
R	R.55.1	650	0.274
R	R.55.2	132	0.056
R	R.55.3	360	0.152
R	R.55.4	204	0.086
R	R.55.5	610	0.257
C.0	C.0.55.1	24	0.086
C.0	C.0.55.2	283	0.119
C.0	C.0.55.3	256	0.108
C.0	C.0.55.4	464	0.195
C.0	C.0.55.5	739	0.311
M.0	M.0.55.1	204	0.086

Mix	Specimen ID	Maximum force (N)	Reported bond stress (MPa)
M.0	M.0.55.2	283	0.119
P	P.55.1	381	0.160
P	P.55.2	801	0.337
P	P.55.3	309	0.130
P	P.55.4	83	0.035
P	P.55.5	93	0.039
A	A.55.1	342	0.144
A	A.55.2	681	0.287

The pilot series showed high variability and includes only *the available* observations. No reclassification or recalculation was applied.

Supplementary Table S4. Individual density measurements.

Mix	Specimen ID	Reported value	Unit
R	R.1	1727.00	kg/m³
R	R.2	1719.00	kg/m³
R	R.3	1762.00	kg/m³
C	C.1	1414.00	kg/m³
C	C.2	1484.00	kg/m³
C	C.3	1602.00	kg/m³
M	M.1	1523.00	kg/m³
M	M.2	1484.00	kg/m³
M	M.3	1473.00	kg/m³
P	P.1	1285.00	kg/m³
P	P.2	1238.00	kg/m³
P	P.3	1270.00	kg/m³
A	A.1	1398.00	kg/m³
A	A.2	1395.00	kg/m³
A	A.3	1578.00	kg/m³
O	O.1	1535.00	kg/m³
O	O.2	1516.00	kg/m³
O	O.3	1484.00	kg/m³

Individual specimen measurements are listed as recorded; replicate numbers are those shown in the table.

Supplementary Table S5. Individual flexural-strength measurements.

Mix	Specimen ID	Reported value	Unit
R	R.1	1.13	MPa
R	R.2	2.18	MPa
R	R.3	2.02	MPa
C	C.1	1.73	MPa
C	C.2	1.97	MPa
C	C.3	2.55	MPa
M	M.1	1.10	MPa
M	M.2	1.59	MPa
M	M.3	1.66	MPa
P	P.1	1.38	MPa
P	P.2	1.05	MPa
P	P.3	1.27	MPa
A	A.1	0.84	MPa

Mix	Specimen ID	Reported value	Unit
A	A.2	1.29	MPa
A	A.3	1.38	MPa
O	O.1	1.90	MPa
O	O.2	2.06	MPa
O	O.3	1.54	MPa

Individual specimen measurements are listed as recorded; replicate numbers are those shown in the table.

Supplementary Table S6. Individual compressive-strength measurements.

Mix	Specimen ID	Reported value	Unit
R	R.1.1	4.81	MPa
R	R.1.2	6.70	MPa
R	R.2.1	8.94	MPa
R	R.2.2	8.14	MPa
R	R.3.1	6.94	MPa
R	R.3.2	6.75	MPa
C	C.1.1	3.89	MPa
C	C.1.2	4.78	MPa
C	C.2.1	4.82	MPa
C	C.2.2	5.51	MPa
C	C.3.1	3.59	MPa
C	C.3.2	3.76	MPa
M	M.1.1	2.08	MPa
M	M.1.2	2.69	MPa
M	M.2.1	2.84	MPa
M	M.2.2	3.20	MPa
M	M.3.1	3.26	MPa
M	M.3.2	3.17	MPa
P	P.1.1	3.86	MPa
P	P.1.2	3.13	MPa
P	P.2.1	3.87	MPa
P	P.2.2	3.47	MPa
P	P.3.1	3.15	MPa
P	P.3.2	4.40	MPa
A	A.1.1	2.76	MPa
A	A.1.2	3.31	MPa
A	A.2.1	4.60	MPa
A	A.2.2	4.21	MPa
A	A.3.1	3.55	MPa
A	A.3.2	3.44	MPa
O	O.1.1	6.39	MPa
O	O.1.2	5.63	MPa
O	O.2.1	4.99	MPa
O	O.2.2	5.14	MPa
O	O.3.1	5.23	MPa
O	O.3.2	5.77	MPa

Individual specimen measurements are listed as recorded; replicate numbers are those shown in the table.
